# Computed Tomography Density and β-Amyloid Deposition of Intraorbital Optic Nerve May Assist in Diagnosing Mild Cognitive Impairment and Alzheimer’s Disease: A ^18^F-Flutemetamol Positron Emission Tomography/Computed Tomography Study

**DOI:** 10.3389/fnagi.2022.836568

**Published:** 2022-03-17

**Authors:** Han Wu, Zhe Lei, Yinghui Ou, Xin Shi, Qian Xu, Keqing Shi, Jing Ding, Qianhua Zhao, Xiuzhe Wang, Xiaolong Cai, Xueyuan Liu, Jingjing Lou, Xingdang Liu

**Affiliations:** ^1^Department of Nuclear Medicine, Huashan Hospital, Fudan University, Shanghai, China; ^2^Department of Nuclear Medicine, Pudong Hospital, Fudan University, Shanghai, China; ^3^Department of Neurology, Zhongshan Hospital, Fudan University, Shanghai, China; ^4^Department of Neurology, Huashan Hospital, Fudan University, Shanghai, China; ^5^Department of Neurology, Shanghai Sixth People’s Hospital Affiliated to Shanghai Jiao Tong University, Shanghai, China; ^6^Department of Neurology, Tenth People’s Hospital affiliated to Tongji University, Shanghai, China

**Keywords:** intraorbital optic nerve, mild cognitive impairment, Alzheimer’s disease, computed tomography, positron emission tomography, ^18^F-flutemetamol, β-Amyloid

## Abstract

**Objective:**

The aim was to study whether the computed tomography (CT) density and β-amyloid (Aβ) level of intraorbital optic nerve could assist in diagnosing mild cognitive impairment (MCI) and Alzheimer’s disease (AD).

**Methods:**

A total of sixty subjects were recruited in our study, including nine normal control (NC) subjects (i.e., 4 men and 5 women), twenty four MCI subjects (i.e., 11 men and 13 women), and twenty seven AD subjects (i.e., 14 men and 13 women). All subjects conducted ^18^F-flutemetamol amyloid positron emission tomography (PET)/CT imaging. Blinded to the clinical information of the subjects, two physicians independently measured and calculated the standardized uptake value ratio (SUVR) of the bilateral occipital cortex, SUVR of the bilateral intraorbital optic nerve, and CT density of the bilateral intraorbital optic nerve by using GE AW 4.5 Workstation.

**Results:**

Between AD and NC groups, the differences of the bilateral intraorbital optic nerve SUVR were statistically significant; between AD and MCI groups, the differences of the left intraorbital optic nerve SUVR were statistically significant. Between any two of the three groups, the differences in the bilateral intraorbital optic nerve density were statistically significant. The bilateral occipital SUVR was positively correlated with the bilateral intraorbital optic nerve SUVR and negatively correlated with the bilateral intraorbital optic nerve density. Bilateral intraorbital optic nerve SUVR was negatively correlated with the bilateral intraorbital optic nerve density. The area under the receiver operating characteristic (ROC) curve of multiple logistic regression was 0.9167 (for MCI vs. NC) and 0.8951 (for AD vs. MCI). The Montreal Cognitive Assessment (MoCA) and Mini-Mental State Examination (MMSE) scores were positively associated with the intraorbital optic nerve density and were negatively associated with the intraorbital optic nerve SUVR. The regression equation of MoCA was *y* = 16.37-0.9734 × *x*_1_ + 0.5642 × *x*_2_-3.127 × *x*_3_ + 0.0275 × *x*_4_; the *R*^2^ was 0.848. The regression equation of MMSE was *y* = 19.57-1.633 × *x*_1_ + 0.4397 × *x*_2_-1.713 × *x*_3_ + 0.0424 × *x*_4_; the *R*^2^ was 0.827.

**Conclusion:**

The CT density and Aβ deposition of the intraorbital optic nerve were associated with Aβ deposition of the occipital cortex and the severity of cognitive impairment. The intraorbital optic nerve CT density and intraorbital optic nerve Aβ deposition could assist in diagnosing MCI and AD.

## Introduction

Alzheimer’s disease (AD) is the most common form of dementia among older adults that affects wide areas of the cerebral cortex and the hippocampus. From a diagnostic perspective, AD is increasingly viewed along a continuum from preclinical AD, through mild cognitive impairment (MCI), to AD dementia (Jack et al., 2011). β-amyloid (Aβ) plaques and neurofibrillary tangles were the characteristic pathologic lesions in the AD brain (Holtzman et al., 2011). Positron emission tomography (PET)/computed tomography (CT) imaging of Aβ in the brain was expected to be useful for improving the accuracy in the diagnosis of AD. However, there is still no effective methods to accurately diagnose MCI and AD; a study showed that Aβ may not be a cause of AD but a consequence of the progression of cognitive impairment (Thomas et al., 2020). Therefore, the application of brain amyloid PET alone may not be sufficient to diagnose MCI and AD, especially in the early stages. It has been demonstrated in a previous study that several patients with AD developed visual anomalies, which were correlated with abnormalities in the optic nerves, such as widespread axonal degeneration and reduction in the thickness of the nerve fiber layer (Hinton et al., 1986). Studies in the past decades reveal that the visual system might be affected by AD, including the optic nerve; the detection of degenerative changes in the optic nerve by medical imaging might be a potential method of diagnosing MCI and AD.

As a part of the central nervous system, the optic nerve travels posteriorly in the orbit, enters the middle cranial fossa *via* the optic canal, connects to the optic cross, and ends at the lateral geniculate body *via* the optic tract to conduct visual impulses. The optic nerve is divided into four parts, namely, the intraocular segment, the intraorbital segment, the intratubular segment, and the intracranial segment; the intraorbital optic nerve was the longest of the four (∼25–30 mm) (Miller, 1996). The fibers of the optic nerve originate from the retinal ganglion cells (RGC). A study showed that there was a functional abnormality of the outer retina concerning the foveal and parafoveal area of the central retina even in the mild stages of AD without visual impairment (Moschos et al., 2012). Previous studies showed that the visual pathway was affected in patients with AD. A study about the retinal nerve fiber layer demonstrated that ocular degeneration in patients with AD and MCI results in decreased thickness of the retinal nerve fiber layer and reduced macular volume in patients with AD and MCI (Gao et al., 2015). Another study about AD transgenic mice showed evidence of molecular, functional, and morphological degenerative changes in the inner retina (Gupta et al., 2016). In another study comparing the optic nerve in patients with AD and normal controls (NCs), a reduction in the number of optic nerve fibers in patients with AD was found (Syed et al., 2005). The previous studies have inspired us that structural and functional optic nerve degeneration should be associated with the ipsilateral occipital visual cortex degeneration in patients with MCI and AD.

Brain amyloid PET/CT was one of the most common and effective modern neuroimaging tools for the diagnosis of MCI and AD (Sevigny et al., 2016). Several fluorine-18-labeled (^18^F) PET tracers, including ^18^F-flutemetamol, have become available for clinical practice and have been incorporated as amyloid pathology biomarkers in the revised research criteria for AD (McKhann et al., 2011). The Aβ deposition in PET images of the AD brain has been demonstrated by many previous studies (Zwan et al., 2017; Cho et al., 2020; Hwang et al., 2021). However, few studies have been conducted on the structural and functional degeneration of the optic nerve in patients with MCI and AD. In Aβ PET brain studies, the standardized uptake value ratio (SUVR) is an effective method that measured the SUV ratio of different brain regions for the semiquantitative analysis (Matsuda et al., 2020); SUVR can reflect the degree of uptake of the radioactive tracers and, consequently, reflect the deposition of Aβ. The Hounsfield unit (HU) was a relatively quantitative measurement of radio density used by radiologists in the interpretation of CT images (Levine et al., 2018); the absorption/attenuation coefficient of radiation within a tissue was used during CT reconstruction to produce a grayscale image. In addition, CT has the advantage of wider availability and significantly lower cost than other neuroimaging methods, such as functional MRI (fMRI), and is still an important tool in clinical practice of neurological diseases in both high-income areas and low- and middle-income areas (Papanicolas et al., 2018; Dieleman et al., 2020). Therefore, the exploration of CT density of the optic nerve could help improve the diagnosis of MCI and AD in low- and middle-income areas and make early treatment available to a wider range of patients with dementia.

To the best of our knowledge, there was no study aimed at Aβ deposition and CT density of the intraorbital optic nerve in patients with MCI and AD. The aim of our research was to study whether CT density and Aβ deposition of the intraorbital optic nerve in ^18^F-flutemetamol PET/CT images could assist in diagnosing MCI and AD. We made a hypothesis that CT density and Aβ deposition of the intraorbital optic nerve would be expected to correlate with the Aβ deposition of the occipital visual cortex and the severity of cognitive impairment.

## Materials and Methods

### Ethics

The study received ethical approval from the Committee for Medical and Health Research Ethics of Huashan Hospital affiliated to Fudan University, Shanghai, China. The clinical registration number is ChiCTR2000035791. The written informed consent was signed by each subject in accordance with the Declaration of Helsinki prior to inclusion in the study. All procedures were conducted in accordance with the institutional regulations and ethical guidelines.

### Participants

Sixty subjects were recruited in this study, including nine NC subjects (i.e., 4 men and 5 women), twenty four MCI subjects (i.e., 11 men and 13 women), and twenty seven AD subjects (i.e., 14 men and 13 women). The NC subjects were recruited from two major communities in Shanghai city; it is necessary to note here that recruiting normal subjects (especially elderly citizens) was a challenge in China due to the lack of scientific education; most of the elderly citizens were so scared of ionizing radiation that they were reluctant to participate in this research. Subjects with MCI and AD were recruited from the outpatient neurology clinics of Huashan Hospital affiliated to Fudan University, Zhongshan Hospital affiliated to Fudan University, and Shanghai Sixth People’s Hospital affiliated to Shanghai Jiao Tong University. The clinical criteria of the National Institute on Aging-Alzheimer’s Association (NIA-AA) workgroups (Jack et al., 2011) were used for the diagnosis of subjects with MCI and AD. All the sixty subjects were tested at inclusion by a standardized neuropsychological battery of tests including the Mini-Mental State Examination (MMSE) and the Montreal Cognitive Assessment (MoCA) for the estimation of cognitive impairment (Folstein et al., 1975; Nasreddine et al., 2005). Subjects were excluded if they had disturbance of myopia, consciousness, delirium, psychosis, severe aphasia, major sensorimotor impairment, and structural brain lesions. All patients regarded themselves as right-handed.

### ^18^F-Flutemetamol Positron Emission Tomography/Computed Tomography Studies

^18^F-flutemetamol PET/CT studies were performed in the Nuclear Medicine Department of Pudong Hospital affiliated to Fudan University, Shanghai, China. All the subjects had an intravenous line while they rested in a quiet and dimly lit room 20 min prior to and 70 min post injection of 200 MBq of ^18^F-flutemetamol (Vizamyl^®^). A normalized PET/CT (Neusoft NeuWise Pro PET/CT) scan was started according to the imaging acquisition guidelines of the Vizamyl^®^,^1^ which recommends a PET scan start time of 60–120 min after Vizamyl^®^ injection. For all participants, all appropriate corrections, including scatter and time-of-flight, were applied with a low-dose CT. Images were reconstructed using the OSEM method (consisting of 16 subsets and 4 iterations) (Nelissen et al., 2009; Vandenberghe et al., 2010). Filtered back-projection reconstruction was used with a slice thickness of 2–4 mm and matrix size of 128 × 128 mm with the pixel size of 2 mm. A full-width half-maximum postsmoothing filter was applied of not more than 5 mm. The duration of the scan lasted 30 min (Nelissen et al., 2009; Vandenberghe et al., 2010). The clinical status was checked before and after the scanning in each participant. Patients were observed for adverse events from the administration of the tracer and were immediately after the PET scan.

### Image Processing and Analysis

Positron emission tomography and CT images were measured by two certified nuclear medicine physicians using the GE AW 4.5 Workstation after passing a subsequent training. The two physicians were blinded to clinical information and independently measured the images using the GE AW 4.5 software according to the training instructions. For PET images, the two physicians independently measured the cortex SUV_max_ of the bilateral cerebellum and the occipital lobe, and the SUV_max_ of the bilateral intraorbital optic nerve and the SUV_max_ values measured by two physicians were averaged. For CT images, two physicians independently measured the mean CT density (HU) of the bilateral intraorbital optic nerve, and the CT density values measured by two physicians were averaged. One side of the cerebellum cortex was used as the ipsilateral reference region to compute the SUVR of the ipsilateral occipital lobe and the intraorbital optic nerve; for instance, the left cerebellum cortex was used as the reference region for left hemispheric measures. Consequently, the left and right SUVR of the bilateral occipital cortex and the intraorbital optic nerve were computed.

### Statistical Analysis

Quantitative variables were described with mean ± SD. Qualitative variables were expressed as absolute and relative frequencies. The Kruskal-Wallis test was used for the comparison of age, education, duration of cognitive impairment, MMSE score, and MoCA score of different groups. The Mann-Whitney *U*-test was used for the comparison of SUVR and CT density between each of the two groups of subjects in NC, MCI, and AD groups; the Bonferroni correction method was used to avoid potential bias due to the small sample size of the NC group; differences were statistically significant if the *p*-value is < 0.017 (corrected: 0.05/3) (Curtin and Schulz, 1998). Simple linear regression was used with the entire sample for the comparison between the occipital cortex SUVR and the intraorbital optic nerve SUVR, between the occipital cortex SUVR and the intraorbital optic nerve CT density, and between the intraorbital optic nerve SUVR and CT density. Multiple logistic regression was used to analyze whether the intraorbital optic nerve SUVR and CT density could assist in distinguishing MCI from NC and AD from MCI. Multiple linear regression was used to explore whether the intraorbital optic nerve SUVR and CT density could predict the MoCA and MMSE scores. Differences were statistically significant if the *p*-value was < 0.05. Analyses were conducted using the IBM SPSS version 26.0 software (SPSS, Chicago, IL, United States).

## Results

### Clinical Characteristics of Study Subjects

Characteristics of the subjects are shown in Table 1. The Kruskal-Wallis test was used for the comparison of age, education, duration of cognitive impairment, MMSE score, and MoCA score of different groups. There was no significant difference in terms of age and duration of disease (between MCI and AD groups). There was a significant difference in terms of MoCA and MMSE scores.

**TABLE 1:** Clinical characteristics of study subjects.

	Normal control	MCI	AD	*p*-value
		
	Mean ± *SD*	Mean ± *SD*	Mean ± *SD*	
Subjects	9	24	27	N/A
Age (years)	60.50 ± 8.90	66.58 ± 8.30	69.00 ± 4.38	0.10
Duration of disease (years)	N/A	2.25 ± 0.89	2.71 ± 1.81	0.64
MoCA (score)	27.50 ± 1.50	21.15 ± 3.55	14.50 ± 2.75	**0.02**
MMSE (score)	28.35 ± 1.06	23.95 ± 3.80	18.00 ± 3.48	**0.03**

*MCI, mild cognitive impairment; AD, Alzheimer’s Disease; MoCA, Montreal Cognitive Assessment; MMSE, Mini-Mental State Examination. p-value below 0.05 were in bold.*

### The Occipital Standardized Uptake Value Ratio, the Intraorbital Optic Nerve Standardized Uptake Value Ratio, and the Intraorbital Optic Nerve Density of Three Groups

**FIGURE 1:**
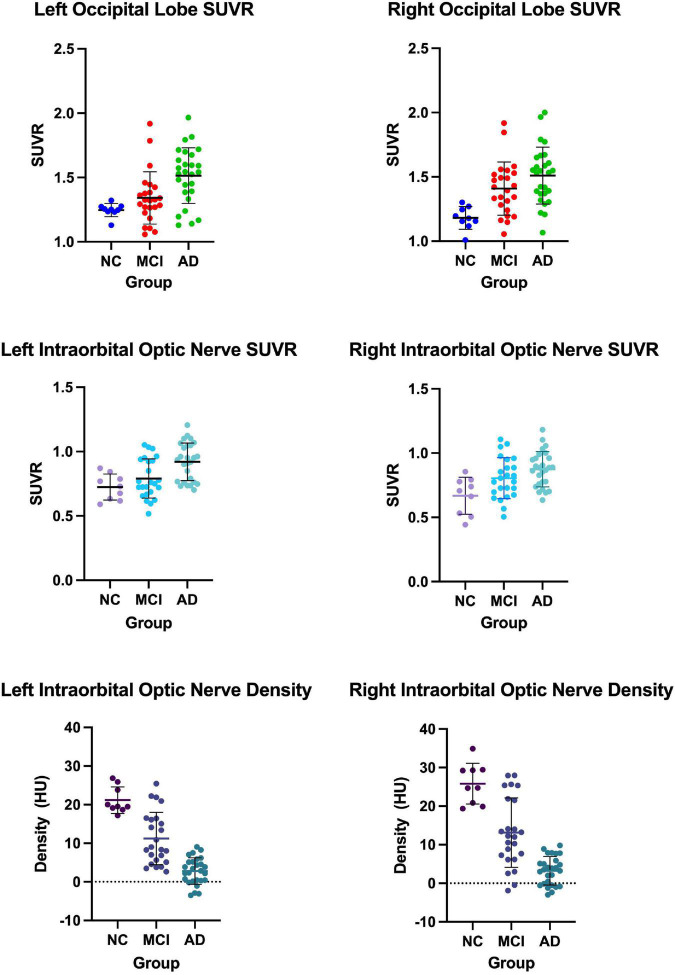
The occipital standardized uptake value ratio (SUVR), the intraorbital optic nerve SUVR, and the intraorbital optic nerve density of three groups.

**FIGURE 2:**
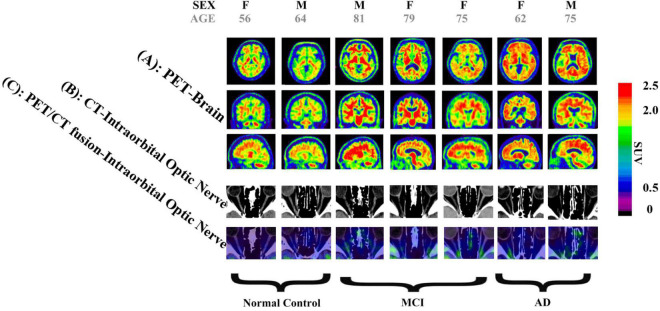
Images of two normal controls (NCs), three subjects with mild cognitive impairment (MCI), and two subjects with Alzheimer’s disease (AD). (A) Brain positron emission tomography (PET) images, showed that: (1) Among images of NC subjects, there is more ^18^F-flutemetamol radioactivity in the white matter than in the gray matter of bilateral occipital lobes, creating clear gray/white matter contrast. (2) Some areas of the gray matter ^18^F-flutemetamol radioactivity of bilateral occipital lobes are as intense as that in the adjacent white matter among MCI subjects. (3) The gray matter ^18^F-flutemetamol radioactivity of bilateral occipital lobes are as intense as that in the adjacent white matter among AD subjects. (B) CT images of bilateral intraorbital optic nerves showed that the CT density of the bilateral intraorbital optic nerves of NC, MCI, and AD subjects was gradually decreased. (C) PET/CT fusion images of the bilateral intraorbital optic nerves showed that: (1) among images of NC subjects, almost no significant ^18^F-flutemetamol radioactivity was observed on the bilateral intraorbital optic nerves; (2) among images of MCI subjects, slight ^18^F-flutemetamol radioactivity was detected on the bilateral intraorbital optic nerves; and (3) among images of AD subjects, ^18^F-flutemetamol radioactivity was detected on the bilateral intraorbital optic nerves.

We measured and calculated the SUVR of the bilateral occipital lobe and the intraorbital optic nerve and measured the mean CT density (HU) of the bilateral intraorbital optic nerve. The values of the abovementioned three groups are shown in Table 2 and Figure 1, and the representative images of subjects with NC, MCI, and AD are shown in Figure 2.

**TABLE 2:** The occipital standardized uptake value ratio (SUVR), the intraorbital optic nerve SUVR, and the intraorbital optic nerve density of three groups.

	Normal control	MCI	AD
	*Mean* ± *SD*	*Mean* ± *SD*	*Mean* ± *SD*
Left occipital lobe SUVR	1.25 ±05	1.34 ± 0.20	1.51 ± 0.22
Right occipital lobe SUVR	1.18 ± 0.09	1.41 ± 0.21	1.51 ± 0.22
Left intraorbital optic nerve SUVR	0.72 ± 0.10	0.79 ±0.15	0.92 ±0.15
Right intraorbital optic nerve SUVR	0.67 ± 0.14	0.81 ± 0.16	0.87 ± 0.14
Left intraorbital optic nerve density (HU)	20.29 ± 4.34	11.23 ± 6.80	2.79 ± 3.45
Right intraorbital optic nerve density (HU)	24.9 ± 6.62	13.1 ± 9.01	3.24 ± 3.68

*MCI, mild cognitive impairment; AD, Alzheimer’s Disease; SUVR, standard uptake value ratio; HU, Hounsfield Unit.*

### Comparison of the Occipital Standardized Uptake Value Ratio, the Intraorbital Optic Nerve Standardized Uptake Value Ratio, and the Intraorbital Optic Nerve Density Between Each of the Two Groups

The Mann-Whitney *U*-test was used for the comparison of occipital SUVR, the intraorbital optic nerve SUVR, and the intraorbital optic nerve CT density between each of the two groups of subjects in NC, MCI, and AD groups. The *p*-values of the Mann-Whitney *U*-test were corrected by the Bonferroni correction between each of the two groups of occipital SUVR, the intraorbital optic nerve SUVR, and the intraorbital optic nerve density, and differences were statistically significant of the *p*-value < 0.017 (corrected: 0.05/3).

Between AD and NC groups, the differences of the bilateral intraorbital optic nerve SUVR were statistically significant. Between AD and MCI groups, the differences of the left intraorbital optic nerve SUVR were statistically significant. Between any two of the three groups, the differences of the bilateral intraorbital optic nerve density were statistically significant. The results are shown in Table 3.

**TABLE 3:** The *p*-value of the occipital SUVR, the intraorbital optic nerve SUVR, and the intraorbital optic nerve density between each of the two groups.

	MCI vs. NC	AD vs. NC	AD vs. MCI
	
	*p*-value	*p*-value	*p*-value
Left occipital lobe SUVR	0.072	**0.002**	**0.002**
Right occipital lobe SUVR	**0.001**	**0.001**	0.023
Left intraorbital optic nerve SUVR	0.116	**0.001**	**0.003**
Right intraorbital optic nerve SUVR	0.079	**0.002**	0.031
Left intraorbital optic nerve Density	**0.004**	**0.001**	**0.001**
Right intraorbital optic nerve Density	**0.001**	**0.001**	**0.001**

*MCI, mild cognitive impairment; AD, Alzheimer’s Disease; NC, normal control; SUVR, standard uptake value ratio.p-values below 0.017 (corrected: 0.05/3) were in bold.*

### Simple Linear Regression Between the Occipital Standardized Uptake Value Ratio and the Intraorbital Optic Nerve Standardized Uptake Value Ratio, Between the Occipital Standardized Uptake Value Ratio and the Intraorbital Optic Nerve Density, and Between the Intraorbital Optic Nerve Standardized Uptake Value Ratio and the Intraorbital Optic Nerve Density

**FIGURE 3:**
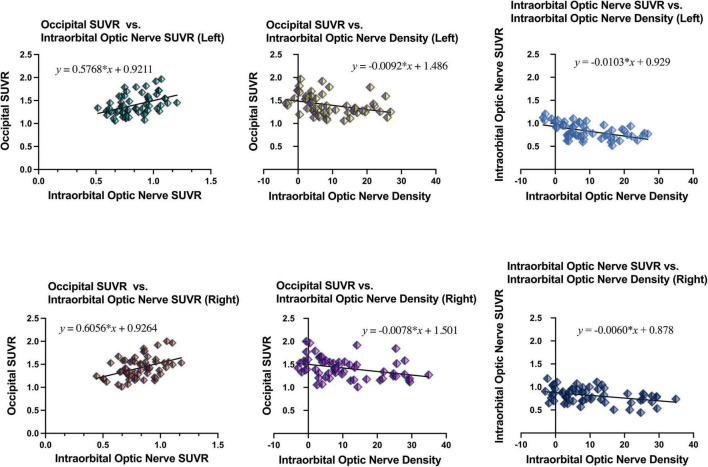
Results of simple linear regression between the occipital SUVR and the intraorbital optic nerve SUVR, between the occipital SUVR and the intraorbital optic nerve density, and between the intraorbital optic nerve SUVR and the intraorbital optic nerve density.

Simple linear regression was used with the entire sample between occipital SUVR and intraorbital optic nerve SUVR, between occipital SUVR and intraorbital optic nerve density, and between intraorbital optic nerve SUVR and intraorbital optic nerve density. The results are shown in Table 4 and Figure 3.

**TABLE 4:** Results of simple linear regression.

	Slope	*p*-value	Equation	*R* ^2^
Occipital SUVR vs. Intraorbital optic nerve SUVR (Left)	0.5768	0.0007	*y* = 0.5768*x + 0.9211	0.1804
Occipital SUVR vs. Intraorbital optic nerve SUVR (Right)	0.6056	0.0006	*y* = 0.6056*x + 0.9264	0.1933
Occipital SUVR vs. Intraorbital optic nerve density (Left)	-0.0092	0.008	*y* = -0.0092*x + 1.486	0.1152
Occipital SUVR vs. Intraorbital optic nerve density (Right)	-0.0078	0.007	*y* = -0.0078*x + 1.501	0.1188
Intraorbital optic nerve SUVR vs. Intraorbital optic nerve density (Left)	-0.0103	0.001	*y* = -0.0103*x + 0.929	0.2623
Intraorbital optic nerve SUVR vs. Intraorbital optic nerve density (Right)	-0.0060	0.003	*y* = -0.0060*x + 0.878	0.1427

*SUVR, standard uptake value ratio.*

In the analysis between occipital SUVR and intraorbital optic nerve SUVR and between occipital SUVR and intraorbital optic nerve density, the occipital SUVR was set as the dependent variable (*y*). Bilateral occipital SUVR (*y*) was positively associated with the bilateral intraorbital optic nerve SUVR (*x*); the regression equation was *y* = 0.5768 × *x* + 0.9211 (left) and *y* = 0.6056 × *x* + 0.9264 (right), respectively. Bilateral occipital SUVR (*y*) was negatively associated with the bilateral intraorbital optic nerve density (*x*), and the regression equation was *y* = -0.0092 × *x* + 1.486 (left) and *y* = -0.0078 × *x* + 1.501 (right), respectively.

In the analysis between the intraorbital optic nerve SUVR and intraorbital optic nerve density, the intraorbital optic nerve SUVR was set as the dependent variable (*y*). Bilateral intraorbital optic nerve SUVR (*y*) was negatively associated with the bilateral intraorbital optic nerve density (*x*); the regression equation was *y* = -0.0103 × *x* + 0.929 (left) and *y* = -0.0060 × *x* + 0.878 (right), respectively.

### Multiple Logistic Regression of the Intraorbital Optic Nerve Standardized Uptake Value Ratio and Computed Tomography Density

Multiple logistic regression was used to analyze whether the intraorbital optic nerve SUVR and CT density could assist in distinguishing MCI from NC and AD from MCI. The groups (i.e., NC, MCI, and AD) of the subjects were set as the dependent variable (*y*); the left and right intraorbital optic nerve SUVR and CT density were set as the independent variables (*x*_1_–*x*_4_, refer to Table 5). The fitting equation of MCI vs. NC was ln[*P*(*y* = 1)/*P*(*y* = 0)] = 1.431-6.737 × *x*_1_-0.2980 × *x*_2_ + 12.68 × *x*_3_ + 0.01946 × *x*_4_; the fitting equation of AD vs. MCI was ln[*P*(*y* = 1)/*P*(*y* = 0)] = 0.1679 + 4.15 × *x*_1_-0.2833 × *x*_2_-0.9103 × *x*_3_-0.1721 × *x*_4_.

**TABLE 5:** Results of multiple logistic regression.

	MCI vs. NC	AD vs. MCI
β0 (Intercept)	1.431	0.1679
β1 (Left Intraorbital optic nerve SUVR, *x*_1_)	-6.737	4.15
β2 (Left intraorbital optic nerve density, *x*_2_)	-0.2980	-0.2833
β3 (Right intraorbital optic nerve SUVR, *x*_3_)	12.68	-0.9103
β4 (Right intraorbital optic nerve density, *x*_4_)	0.01946	-0.1721
Fitting equation	ln[*P*(*y* = 1)/*P*(*y* = 0)] = 1.431-6.737*x_1_-0.2980*x_2_ + 12.68*x_3_ + 0.01946*x_4_	ln[*P*(*y* = 1)/*P*(*y* = 0)] = 0.1679 + 4.15*x_1_–0.2833*x_2_ -0.9103*x_3_-0.1721*x_4_

*MCI, mild cognitive impairment; AD, Alzheimer’s Disease; NC, normal control; SUVR, standard uptake value ratio.*

**FIGURE 4:**
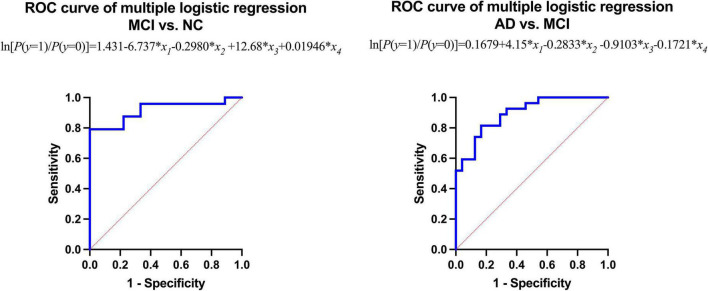
Receiver operating characteristic (ROC) curve of multiple logistic regression.

The area under the curve (AUC) of the receiver operating characteristic (ROC) of multiple logistic regression was 0.9167 (for MCI vs. NC) and 0.8951 (for AD vs. MCI), respectively, and the *p*-values of AUC were 0.0003 (for MCI vs. NC) and 0.0001 (for AD vs. MCI), respectively. The results are shown in Tables 5, 6 and Figure 4.

**TABLE 6:** Results from receiver operating characteristic (ROC) analysis of multiple logistic regression.

	MCI vs. NC	AD vs. MCI
Area under the ROC curve (AUC)	0.9167	0.8951
*p*-value of AUC	0.0003	0.0001
Cut off	0.7209	0.5505
Sensitivity (%)	79.17	81.48
Specificity (%)	88.89	83.33

*MCI, mild cognitive impairment; AD, Alzheimer’s Disease; NC, normal control.*

### Multiple Linear Regression Between Neuropsychological Tests Scores and the Intraorbital Optic Nerve Standardized Uptake Value Ratio and the Intraorbital Optic Nerve Density

**FIGURE 5:**
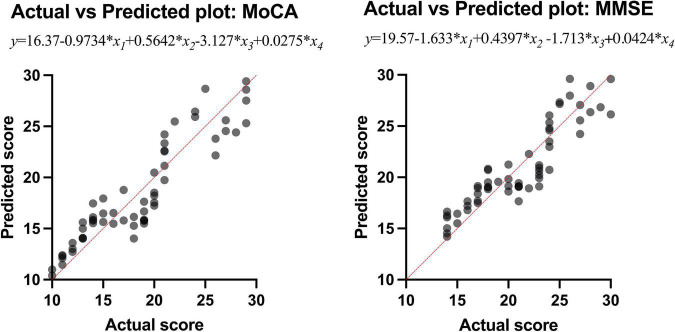
The actual vs. predicted plots of multiple linear regression.

Multiple linear regression was used to explore whether the intraorbital optic nerve SUVR and CT density could predict the MoCA and MMSE scores. The MoCA and MMSE scores were set as the dependent variable (*y*), and the left and right intraorbital optic nerve SUVR and CT density were set as the independent variables (*x*_1_–*x*_4_, refer to Table 7). The MoCA and MMSE scores were positively associated with the intraorbital optic nerve density and negatively associated with the intraorbital optic nerve SUVR. The regression equation of MoCA was *y* = 16.37-0.9734 × *x*_1_ + 0.5642 × *x*_2_-3.127 × *x*_3_ + 0.0275 × *x*_4_; the *R*^2^ was 0.848. The regression equation of MMSE was *y* = 19.57-1.633 × *x*_1_ + 0.4397 × *x*_2_-1.713 × *x*_3_ + 0.0424 × *x*_4_; the *R*^2^ was 0.827. The actual vs. predicted plots are shown in Figure 5.

**TABLE 7:** Results of multiple linear regression.

	MoCA	MMSE
β0 (Intercept)	16.37	19.57
β1 (Left intraorbital optic nerve SUVR, *x*_1_)	−0.9734	−1.633
β2 (Left intraorbital optic nerve density, *x*_2_)	0.5642	0.4397
β3 (Right intraorbital optic nerve SUVR, *x*_3_)	−3.127	−1.713
β4 (Right intraorbital optic nerve density, *x*_4_)	0.0275	0.0424
Fitting equation	*y* = 16.37−0.9734*x_1_ + 0.5642*x_2_−3.127* *x*_3_ + 0.0275*x_4_	*y* = 19.57−1.633*x_1_ + 0.4397*x_2_ −1.713*x_3_ +0.0424*x_4_
*R* ^2^	0.848	0.827

*MoCA, Montreal Cognitive Assessment; MMSE, Mini-Mental State Examination; SUVR, standard uptake value ratio.*

## Discussion

The AD-related degeneration of the optic nerve is characterized by irreversible structural and functional changes. Some studies have reported the loss of large diameter axons (Hinton et al., 1986), while others have suggested that optic nerve axons are lost in small size (Syed et al., 2005). In another case-control study comparing the optic nerve in patients with AD and NCs, it was found that there was a reduction in the number of optic nerve fibers in patients with AD, with a threefold greater odds ratio for a larger optic cup-to-disc ratio in patients with AD (Danesh-Meyer et al., 2006). These above studies aimed at the intraocular segment of the optic nerve, but we focused on structural and functional changes of the intraorbital optic nerve in this study.

In our study, the comparison of the occipital SUVR between each of the two groups showed that the differences were statistically significant, except for the left occipital SUVR between MCI and NC (*p* = 0.218) and the right occipital SUVR between AD and MCI (*p* = 0.068). We initially thought that the comparisons between each of the two groups of the occipital SUVR would be statistically significant. Considering that the sample size was not large enough might be the possible reason why the results were different than expected; especially, the sample size of the NC group was small due to the difficulty in recruiting normal subjects. A previous study using ^18^F-flutemetamol PET showed that the differences of both the left and right occipital SUVR between subjects with MCI and AD were statistically significant with high efficacy (bao et al., 2021).

To our surprise, the differences of bilateral intraorbital optic nerve SUVR between MCI and NC groups were not statistically significant, while the differences were statistically significant between AD and NC groups. It means that there was significant amyloid deposition in bilateral intraorbital optic nerves of patients with AD, not of patients with MCI. This finding was consistent with a recent study that indicated that amyloid deposition might be the result rather than the cause of neurodegeneration (Thomas et al., 2020). In addition, the differences of the intraorbital optic nerve SUVR between AD and MCI groups were statistically significant on the left only, consistent with the results of the occipital SUVR between AD and MCI groups (also statistically significant on the left only). This might imply that the deposition of amyloid in the intraorbital optic nerve was consistent with that of the occipital lobe in patients with AD; whether the deposition of amyloid in the intraorbital optic nerve was associated with other cerebral regions needs further research to demonstrate.

The differences of the bilateral intraorbital optic nerve density were statistically significant between any two of the three groups. This might indicate that bilateral intraorbital optic nerve degeneration begins at the MCI stage, and CT density reflects the degree of optic nerve degeneration. A study showed that there was a difference between optic nerve volumes of subjects with AD and control subjects, but there was no correlation between the optic nerve volume and cerebral volume in patients with AD (Kusbeci et al., 2015). This meant that degenerative changes in the optic nerve possibly tend to develop independently rather than in parallel with degenerative changes in the brain. A previous study about the human visual pathway demonstrated that the spread of neurodegeneration may be independent of the neurotransmission machinery (You et al., 2019). If this hypothesis could be confirmed by more studies in the future, visual degeneration might become an independent marker to diagnose cognitive impairment and dementia.

In this study, the results of simple linear regression showed that the bilateral occipital SUVR was positively associated with the bilateral intraorbital optic nerve SUVR, the bilateral occipital SUVR was negatively associated with the bilateral intraorbital optic nerve density, and the bilateral intraorbital optic nerve SUVR was negatively associated with the bilateral intraorbital optic nerve density. Similar to the visual cortex, it was no surprise that the occipital SUVR was associated with the structural and functional degenerative changes of the optic nerve, as well as the embryological ties of the neuroretina and brain structures stated in a study (Coppola et al., 2015). The negative association between the occipital SUVR and intraorbital optic nerve density was an exciting finding to us, as the CT density of the optic nerve was much easier to measure compared with the measurement of cerebral amyloid deposition. Also, the CT scan is more accessible than fMRI and PET brain scan, especially in low- and middle-income countries and areas. According to the above results, the lower intraorbital optic nerve density was corresponded to the higher occipital SUVR and intraorbital optic nerve SUVR and indicated a higher level of amyloid deposition in the intraorbital optic nerve and in the brain. However, the intraorbital optic nerve density should not only be associated with cerebral amyloid deposition and cognitive impairment. Many physiological or pathological factors may influence intraorbital optic nerve density, such as age, nutritional status, daily light hours, and daily sleep duration (Woon et al., 1995; Chapman et al., 2012; Mentek et al., 2018). Further studies with larger sample size are needed to study the relationship between the optic nerve density and other influencing variables.

The results of multiple logistic regression showed that the intraorbital optic nerve SUVR and CT density could assist in distinguishing MCI from NC and AD from MCI. The fitting equation of MCI vs. NC was ln[*P*(*y* = 1)/*P*(*y* = 0)] = 1.431-6.737 × *x_1_-*0.2980 × *x*_2_ + 12.68 × *x*_3_ + 0.01946 × *x*_4_; the fitting equation of AD vs. MCI was ln[*P*(*y* = 1)/*P*(*y* = 0)] = 0.1679 + 4.15 × *x*_1_-0.2833 × *x*_2_-0.9103 × *x*_3_-0.1721 × *x*_4_. In the fitting equations, *x*_1_–*x*_4_ represent the left intraorbital optic nerve SUVR, the left intraorbital optic nerve density, the right intraorbital optic nerve SUVR, and the right intraorbital optic nerve density, respectively. The ROC analysis of multiple logistic regression showed that the AUC was 0.9167 (for MCI vs. NC) and 0.8951 (for AD vs. MCI), which suggested that the efficiency and efficacy in distinguishing MCI from NC and AD from MCI were relatively high.

The results of multiple linear regression showed that the intraorbital optic nerve SUVR and CT density were associated with MoCA and MMSE scores, demonstrating that the Aβ deposition and CT density of intraorbital optic nerve were correlated with the severity of cognitive impairment. A study showed that postmenopausal women who had large cup-to-disc ratio without glaucoma or ocular hypertension exhibited lower global cognitive function (Vajaranant et al., 2019). Another study showed that the decrease of the coronal optic nerve sheath diameter was associated with postoperative cognitive decline (Zhang et al., 2021). These studies demonstrated that multiple structural degeneration of the optic nerve was associated with cognitive impairment. The regression equation of MoCA was *y* = 16.37-0.9734 × *x*_1_ + 0.5642 × *x*_2_-3.127 × *x*_3_ + 0.0275 × *x*_4_; the *R*^2^ was 0.848. The regression equation of MMSE was *y* = 19.57-1.633 × *x*_1_ + 0.4397 × *x*_2_-1.713 × *x*_3_ + 0.0424 × *x*_4_; the *R*^2^ was 0.827. The regression equations and their *R*^2^ indicated that the intraorbital optic nerve SUVR and CT density could predict MoCA and MMSE scores with a relatively high ability. Figure 5 showed that the scatters of the predicted MoCA and MMSE scores were mostly around the red straight line and demonstrated that the regression models with intraorbital optic nerve SUVR and CT density could predict MoCA and MMSE scores and further predict the severity of cognitive impairment. This means that a high likelihood of cognitive impairment should be noted if a decreased intraorbital optic nerve density was found on cranial CT images, and further examination like brain amyloid PET should be conducted to confirm the cognitive impairment.

In this study, we examined the relationship between occipital SUVR and intraorbital optic nerve SUVR and its CT density, demonstrated the assisting ability of the optic nerve SUVR and CT density in diagnosing MCI and AD, and found intraorbital optic nerve SUVR and its CT density could help predict MoCA and MMSE scores and further predict the severity of cognitive impairment. We have made our efforts to fill the gap in the structural and functional changes of the optic nerve and the association between the optic nerve and the cerebral visual cortex. In addition, the potential application of intraorbital optic nerve CT density could reduce the cost of diagnosis of MCI and AD. Compared with traditional imaging methods for diagnosing MCI and AD such as fMRI and PET, CT was less expensive and more accessible, especially in low- and middle-income areas. Therefore, this might enable early diagnosis of AD to reach more people and expand the coverage of precise treatment of patients with AD.

There were several limitations in our study. First, the sample size was not large enough, especially, the sample size of the NC group was small due to the difficulty in recruiting normal subjects; studies with larger samples covering a wider age range are needed in the future to confirm the results of this study. Second, further and deeper studies (especially animal experiments based on the cellular and molecular mechanisms) aimed at the association between occipital Aβ deposition and intraorbital optic nerve density are strongly needed to clearly demonstrate and clarify the mechanisms.

## Conclusion

The CT density and Aβ deposition of the intraorbital optic nerve were associated with Aβ deposition of in occipital cortex and the severity of cognitive impairment. The intraorbital optic nerve CT density and intraorbital optic nerve Aβ deposition could assist in diagnosing MCI and AD.

## Data Availability Statement

All data in this study are clinical data. According to the requirements of the ethics committee of Huashan Hospital, Zhongshan Hospital, and Shanghai Sixth People’s Hospital, data is only to be made available via a request to the authors, and after requesting approval from the authors’ local ethics committee. Requests to access the datasets should be directed to JL and XdL (1436150464@qq.com, xingdliu@fudan.edu.cn).

## Ethics Statement

The studies involving human participants were reviewed and approved by the Committee for Medical and Health Research Ethics of Huashan Hospital affiliated to Fudan University, Shanghai, China. The patients/participants provided their written informed consent to participate in this study.

## Author Contributions

HW recruited the subjects and wrote this manuscript. ZL and YO processed the data and assisted in writing the manuscript. XS, QX, and KS injected the subjects with the tracer and acquired the images. JD, QZ, XW, XC, and XyL helped to recruit subjects. JL helped design the research. XdL designed the research and provided funding. All authors contributed to the article and approved the submitted version.

## Conflict of Interest

The authors declare that the research was conducted in the absence of any commercial or financial relationships that could be construed as a potential conflict of interest. JZ declared a shared parent affiliation with XL at the time of review.

## Publisher’s Note

All claims expressed in this article are solely those of the authors and do not necessarily represent those of their affiliated organizations, or those of the publisher, the editors and the reviewers. Any product that may be evaluated in this article, or claim that may be made by its manufacturer, is not guaranteed or endorsed by the publisher.

## Footnotes

1
https://www.accessdata.fda.gov/drugsatfda_docs/label/2017/203137s008lbl.pdf
